# Does Cricoid Pressure Increase the Laryngoscopy Force During Rapid Sequence Induction?—A Randomized Study

**DOI:** 10.5152/TJAR.2022.21166

**Published:** 2023-02-01

**Authors:** Gourav Mittal, Divya Jain, Shalvi Mahajan, Goverdhan Dutt Puri, Jaspreet Singh, Ashok Kumar

**Affiliations:** 1 Department of Anaesthesia and Intensive Care, Post Graduate Institute of Medical Education & Research, Chandigarh, India; 2 Semiconductor Laboratory, Mohali, Punjab, India; 3 National Institute of Nursing Education, Post Graduate Institute of Medical Education & Research, Chandigarh, India

**Keywords:** Anaesthesia, anaesthesia complications, airway, general anaesthesia, intubation, rapid sequence induction

## Abstract

**Objective:**

Cricoid pressure, a manoeuver used to prevent pulmonary aspiration during rapid sequence induction, can result in deterioration of laryngeal view and increased haemodynamic changes. Its effect on laryngoscopy force remains unevaluated. The study aimed to assess the impact of cricoid pressure on laryngoscopy force and intubation characteristics during rapid sequence induction.

**Methods:**

Seventy American Society of Anaesthesiologists I/II patients, both sexes, aged 16-65, having non-obstetric emergency surgery were randomly assigned to the cricoid group, which received 30 N cricoid pressure during rapid sequence induction, and the sham group, which received 0 N pressure. Propofol, fentanyl, and succinylcholine were used to produce general anaesthesia. The primary outcome was the peak force of laryngoscopy. Secondary outcomes were the laryngoscopic view, time to execute endotracheal intubation, and intubation success rate.

**Results:**

With the application of cricoid pressure, the peak forces of laryngoscopy increased significantly, with a mean difference (95% CI) of 15.5 (13.8-17.2) N. With and without CP, the mean peak forces were 40.758 (4.2) and 25.2 (2.6) N, respectively, *P*  < .001. Without cricoid pressure, the intubation success rate was 100%, compared to 85.7% with cricoid pressure, *P*  = .025. The proportions of CL1/2A/2B patients with and without cricoid pressure were 5/23/7 and 17/15/3, respectively, with *P*  = .005. With cricoid pressure, there was a considerable increase in intubation duration, with a mean difference (95% CI) of 24.4 (2.2-19.9) seconds.

**Conclusion:**

Cricoid pressure increases peak forces during laryngoscopy, resulting in worse intubation characteristics. This demonstrates the need of exercising care while performing this manoeuver.

**Main Points**

- Cricoid pressure is often applied to prevent aspiration.
- It’s use resulted in significant increase in peak pressure applied during laryngoscopy.
- This warrants caution while applying cricoid pressure.

## Introduction

Cricoid pressure (CP) is a manoeuver proposed by Sellick^1^ in 1961 to prevent regurgitation of gastric contents while performing endotracheal intubation during rapid sequence induction in emergency settings. This age-old practice of applying CP has been surrounded with controversies regarding its efficacy in preventing regurgitation^2^ and potential risks associated with the technique.^3,4^

Few studies have reported difficulty in mask ventilation, deterioration of laryngeal view, and difficulty in intubation with the application of cricoid force.^5-8^

Laryngoscopy and intubation can induce haemodynamic perturbations in the form of tachycardia and hypertension.^9^ Factors which result in increased force during laryngoscopy can accentuate these haemodynamic fluctuations, resulting in adverse cardiac events in susceptible individuals. The force of laryngoscopy is dependent on various patient- and performer-related factors. Patient factors include the age, body mask index (BMI), and presence of bucked or protruding teeth, while the performers factors include the experience and technique of the laryngoscopist.^10^ Therefore, it can be implied that factors which increase the difficulty of laryngoscopy can result in greater force of laryngoscopy. Previous studies reporting force of laryngoscopy observed greater peak force in patients with poor laryngeal view. The worsening of the laryngeal view with CP may warrant increase in the force of laryngoscopy.

Till date, the effect of CP on laryngoscopy force remains unevaluated. We hypothesized that the application of CP would significantly increase the peak force of laryngoscopy. The primary objective was to measure the peak force generated during laryngoscopy with Macintosh laryngoscope in patients with and without CP. Time taken to intubate, glottic visualization, and haemodynamic response were recorded as secondary outcomes.

## Methods

### Study Design

This prospective randomized trial was conducted in the tertiary care Hospital of India after approval from the Postgraduate Institute of Medical Education and Research, Chandigarh, India (NK/6249/Study/131) dated May 8, 2020, and written informed consent from all study participants. The trial was commenced after being registered with Clinical Trial Registry (CTRI/2020/06/0025562). 

### Inclusion and Exclusion Criteria

Seventy American Society of Anaesthesiologists (ASA) I and II patients between 16 years to 65 years of either sex were enroled in this prospective randomized trial. Patients with anticipated difficult airway, cardiovascular disease, symptomatic gastroesophageal reflux or reactive airway disease, and ASA >3 were excluded.

### Study Protocol

In the operation theatre, standard monitors including pulse oximeter, non-invasive blood pressure, electrocardiograph, and capnograph (Aestiva 5TM 7900, Datex Ohmeda, USA) were applied. All patients were preoxygenated with 100% oxygen for 3 minutes. General anaesthesia was induced with propofol 2-2.5 mg kg^−1^ and fentanyl 1-2 μg kg^−1^. Succinylcholine 2 mg kg^−1^ was given for muscle relaxation. The patient was placed in sniffing position (extension of the head and flexion of the neck). Cricoid pressure or sham pressure was applied after induction of anaesthesia using 2 hand technique according to the group allocation. Patients were randomized using computer-generated random numbers into 2 groups in which tracheal intubation was performed with and without CP. In the CP group, 30 N pressure was applied by the trained resident, while 0 N was applied in the sham group. The allocation to the group was concealed in sequentially labelled opaque envelopes.

Before the commencement of the trial, all the residents and paramedical staff posted in the emergency operation theatre were trained to apply the CP on a mannequin using a 50 mL syringe model for 3 consecutive days. They were instructed to reduce the volume of an air filled obtruded 50-mL syringe to 33 mL to apply 30 N pressure. This method of training to apply cricoid pressure had been successfully demonstrated by Kopka et al^1^ ^1^ who had tested this using custom-made weights and a regularly serviced electronic scale (Seca model 727). Only trained residents were allowed to perform the CP. In the sham group, a hand was placed on the cricoid cartilage without applying any pressure for blinding.

**Figure 1.**
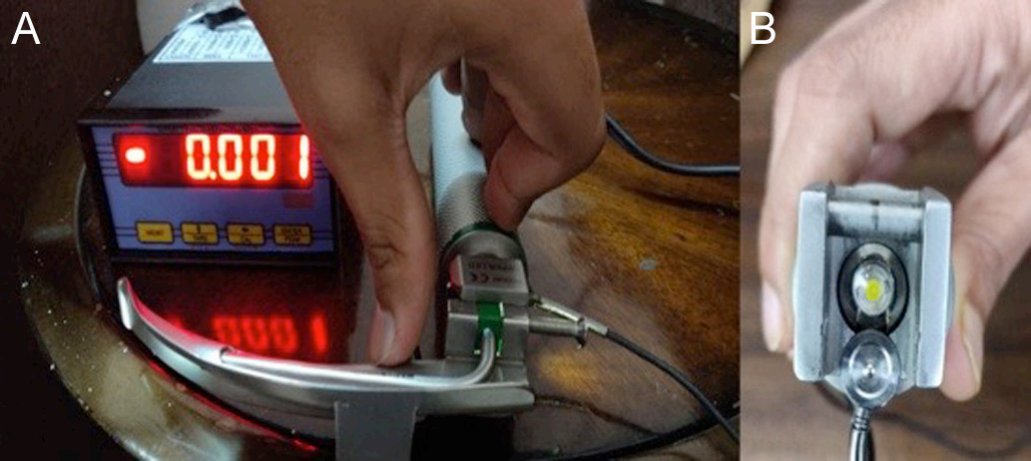
Laryngoscope with stain gauge sensor.

A specially designed laryngoscope equipped with strain gauge sensor placed between the blade and the handle to measure the force parallel to the axis of the handle was used to measure the peak force of laryngoscopy. It had a small display unit, which showed the peak force (Figure 1).

Laryngoscopy and endotracheal intubation were performed by 2 investigators (GM, SM) with an experience of more than 100 intubations each. The screen of the display unit was kept facing the blinded observer, who recorded the parameters. 

### Parameters Recorded

The time taken to intubation was measured from the time the laryngoscope blade was inserted in the patient’s mouth till the first capnograph trace was seen. The grading of the laryngeal opening was done by the laryngoscopist using Cormack–Lehane (CL) grading. Peak force of laryngoscopy was recorded as primary outcome. Impulse force, laryngoscopic view obtained by CL grade, time taken to intubate, number of attempts taken to intubate, manoeuvers used and haemodynamic response such as heart rate (HR) and mean arterial pressure (MAP), and any desaturation (oxygen saturation <95%) observed were recorded as secondary outcomes.

### Statistical Analysis

Sample size calculation was based on the previous study by Bucx et al^12^ who observed applied peak force of 35 N (standard deviation: 12) using Macintosh blade in normal airway patients. Anticipating a 30% increase as clinically relevant increase in force, we calculated the sample size. For an alpha of 0.05 and power of 0.8, we required a sample size of 32 patients in each group. To account for the dropouts, 70 patients would be enroled in the study.

The statistical analysis was carried out using Statistical Package for Social Sciences version 22.0 software (IBM Corp.; Armonk, NY, USA). Mean and medians were calculated for all quantitative variables. Normality of data was checked by Kolmogorov–Smirnov tests of normality. For normally distributed data, the means of 2 groups was compared using *t*-test. For skewed data, Mann–Whitney test was applied. Qualitative or categorical variables were described as frequencies and proportions. For comparison of tim-related variables, repeated measure analysis of variance (ANOVA) was used. All statistical tests were 2-sided and were performed at a significance level of α = 0.05.

## Results

**Figure 2.**
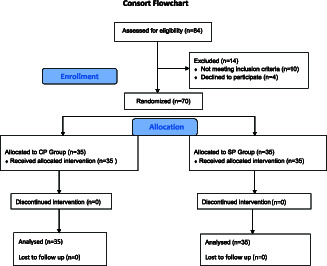
Consort flowchart.

Eighty-four patients were assessed for eligibility out of which 10 did not meet the inclusion criteria and 4 declined consent. A total of 70 patients were randomized and analyzed (Figure 2). The demographic and intraoperative data were comparable between the 2 groups (Table 1).

### Force of Laryngoscopy

There was a significant increase in the peak forces of laryngoscopy with the use of CP with a mean difference (95% CI) of 15.5 (13.8-17.2) N. The mean peak forces with and without CP were 40.758 (4.2) and 25.2 (2.6) N, respectively, *P*  < .001. 

### Intubation Parameters

The intubation success rate was 100% without CP compared to 85.7% with CP, *P*  = .025. The proportion of patients with CL1/2A/2B with and without CP were 5/23/7 and 17/15/3, respectively, *P*  = .005. Two patients (5.7%) in CP group and 3 patients (8.5%) without CP group were intubated in the second attempt (*P*  = .65). Bougie was used in 6 patients (17%) in CP group and 2 patients (5.7%) in without CP group (*P*  = .139). Cricoid pressure resulted in a significant increase in the time taken for intubation with a mean difference (95% CI) of 24.4 (2.2- 19.9) seconds (Table 2).

### Haemodynamic Changes

**Figure 3.**
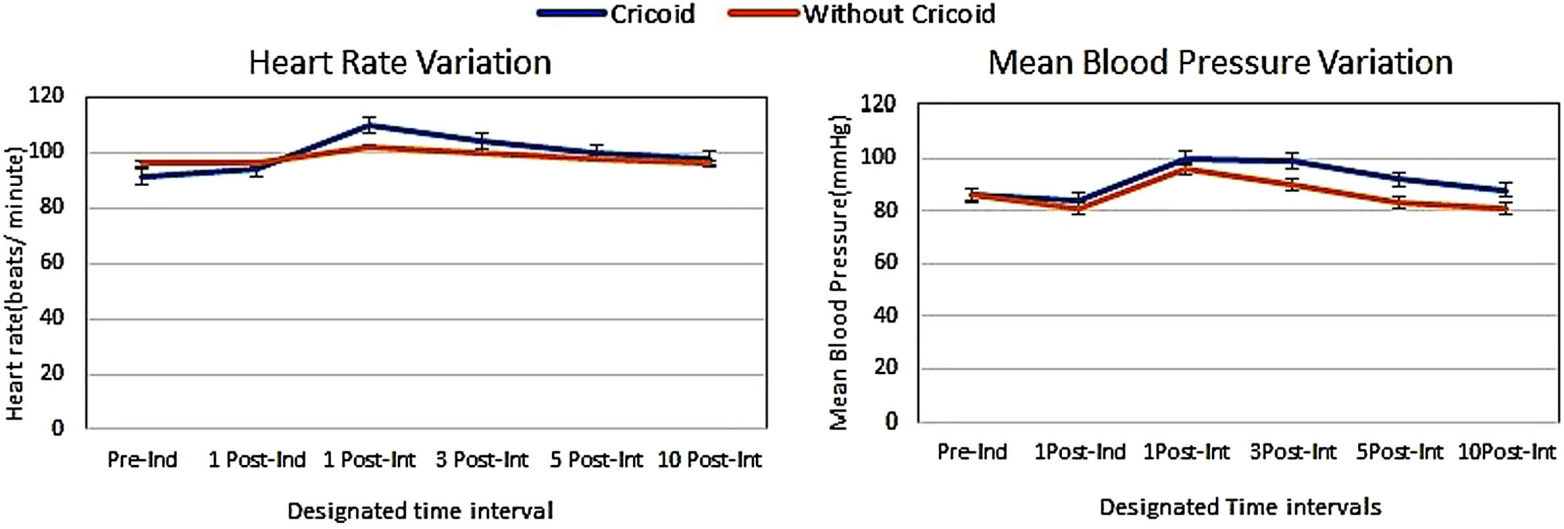
Haemodynamic changes during anaesthesia induction.

There was a significant increase in the heart rate (*P*  = .035) in the CP group at 1 minute of post -ntubation, while increase in MAP was seen in the CP group at 1 minute (*P*  = .000) and 3 minutes (*P*  = .040) post-intubation (Figure 3).

### Adverse Events

Minimum desaturation (SpO_2_ below 95%) was seen in 16 patients in CP group and 4 patients without CP. Minimal trauma was seen in 1 patient in both the groups.

## Discussion

Ours is the first study to document and quantify the increase in the force of laryngoscopy in patients whom CP is applied. Bucx et al^12^ were one of the initial researchers to demonstrate the force required during laryngoscopy. Later, Hastings et al^13^ further elaborated that laryngoscopy is a complex procedure involving not just axial but torque forces as well. Their study highlighted that the axial force was a predominant force during laryngoscopy, whereas torque and other forces had minimal contribution to the force of laryngoscopy. An important observation in these studies was the increased force of laryngoscopy in patients with poor glottic view. However, none of the previous studies measuring the force of laryngoscopy included patients in whom CP was applied. 

During CP application, the tissues that surround the vallecula are forced into closer contact with the laryngoscope blade and the pressure exerted on the laryngoscopy blade can be expected to increase. Another reason for the increase in peak force can be the hampered glottic visibility with the application of CP. To attain an optimal view of the glottis aperture for endotracheal intubation, greater axial force is required. This seems to be in line with the earlier reports by Hasting et al.^13^

So far, there have been numerous published articles, with contradictory results, reporting the effect of CP on laryngeal view and tracheal intubation.^14^ Vanner et al^15^ showed improvement in the laryngeal view with CP. Contrary to these findings, a study evaluating the effect of CP on laryngoscopic view through endoscopic photography found marked deterioration of laryngeal view in 20% of the patients.^16^ Noguchi et al^17^ designed a study to compare the gum elastic bougie and stylet during endotracheal intubation with CP and found significant worsening the laryngeal view with CP.

In the present study, we observed significant deterioration of glottic view in the CP group with application of 30 N force. Haslam et al^16^ reported complete loss of the glottic view in a group of patients with 30 N force.

In contrast to the previous studies, we could not report the change in the glottis view with CP as laryngoscopy in the CP group was performed after the application of the CP.

Saghaei et al^18^ showed that the compression of laryngeal and perilaryngeal structures with the application of CP leads to stimulation of the autonomic nervous system resulting in increase in HR and BP, which is further accentuated during induction of anesthesia with laryngoscopy and intubation. Although we observed significant increase in the haemodynamic response during laryngoscopy and intubation in the CP group, there was no significant increase in HR or MAP postinduction of anaesthesia following application of CP. 

Correct application of CP has also been a matter of concern. In this study, 30 N is considered sufficient enough to occlude the esophagus and reduce the risk of pulmonary aspiration. However, it is difficult for practitioners to accurately estimate this force in everyday practice. Various methods investigated to train the staff on the correct application of CP include the weighing scale method, simulation-based training, and use of a 50 mL syringe.^18^,^19^ Kopka et al^11^ and Flucker et al^20^ suggested the use of a 50‐mL syringe as a simple and economical method to be used in everyday clinical practice.

The findings of our study highlight another implication of the CP in the form of increased peak force of laryngoscopy. The increased forced combined with the increased duration of laryngoscopy can be hazardous in patients prone to cardiac complications.

The results of our study should be interpreted in the light of some limitations. Firstly, the laryngoscope was equipped to measure only the axial force. However, in the hands of trained personal, the effect of torque is minimal. The intubation was performed by the senior resident who had more than 4-5-year experience in anaesthesia. Secondly, as already discussed application of CP is relatively subjective, despite the training of the staff prior to the study. The skin compliance of a manikin is not the same as the patients; therefore, application of 30 N force might not be transmitted equally in a manikin and a patient. The use of a sensor to monitor 30 N force would have been ideal. Lastly, complete blinding of the laryngoscopist to the CP manoeuver was not possible despite the use of sham technique, in which the trained personnel placed a hand on the cricoid cartilage to mimic CP application.

## Conclusion

To conclude, our findings show significant increase in the peak forces during laryngoscopy with application of CP along with deterioration of intubation parameters. This highlights the need for caution while using this maneuver in susceptible individuals. 

## Figures and Tables

**Table 1.** Demographic Data of Patients

Parameter	CP group (n = 35)	SP group (n = 35)	*P*
Age (years)	44.61 (16.6)	41 (13.8)	.32
Gender (male:female)	23/12	25/10	.61
Height (cm)	162.5 (6.5)	160.6 (8.8)	.30
Weight (kg)	59 (11.2)	61.6 (10.8)	.32
ASA status (I/II/III)	16/19	21/14	.23
MMP (I/II/III)	15/17/3	17/14/4	.75
Neck circumference (cm)	26.1 (2.4)	27 (2.3)	.11
Thyromental distance (cm)	7 (0.2)	6.8 (0.3)	.10

Data are expressed mean (SD) or absolute numbers. *P* < .05 is statistically significant.

n, number of patients; CP, cricoid pressure; SP, sham pressure; ASA, American Society of Anaesthesiologists; MMP, modified Mallampati grade.

**Table 2.** Intubation Characteristics in 2 Groups

Intubation Characteristics	Group CP (n = 35)	Group SP (n = 35)	Mean Difference (95% CI)
Peak forces (N)	40.7 (4.2)	25.2 (2.6)	15.5 (13.8-17.2)
Intubation success rate	30 (85.7%)	35 (100%)	14.3 (1.5%-29.3%)
Laryngoscopic view (CL 1/2a/2b/3)	5/23/7	17/15/3	
Time taken to intubate (s)	48.2 (12.5)	23.7 (5.9)	24.4 (2.2-19.9)

Data are expressed as mean (SD), absolute numbers, or number (percentage).

n, number of patients; CP, cricoid pressure; SP, sham pressure; CL, Cormack–Lehane grading.
